# Effects of limb-specific visual occlusion on bimanual reach-to-grasp coordination in an immersive virtual reality environment

**DOI:** 10.3389/fpsyg.2026.1884831

**Published:** 2026-08-19

**Authors:** Guilherme D. Martins, Bryce Sprecher, Andrea H. Mason

**Affiliations:** Department of Kinesiology, University of Wisconsin-Madison, Madison, WI, United States

**Keywords:** bimanual coordination, kinematics, motor control, reach-to-grasp, sensorimotor integration, virtual reality, visual feedback, visuomotor control

## Abstract

**Introduction:**

Visual feedback plays an important role in bimanual coordination, but the specific contribution of visual information from each limb during symmetric bimanual reach-to-grasp movements remains unclear. This study examined how selective visual occlusion of one or both hands influences movement coordination in an immersive virtual reality environment.

**Methods:**

Nineteen right-handed participants performed symmetric bimanual reach-to-grasp movements under four visual feedback conditions: both hands visible (BV), left hand absent (LA), right hand absent (RA), and both hands absent (BA). Movement initiation, movement termination, movement time, peak velocity, time after peak velocity, path length, lateral trajectory deviation, and grasp success were analyzed.

**Results:**

Movement initiation remained synchronized across visual conditions, whereas movement termination, movement time, trajectory characteristics, and grasp success were influenced by selective visual occlusion. Peak velocity showed condition-dependent differences between the hands, while movement time increased primarily for the occluded hand under unilateral visual occlusion. The non-dominant hand consistently exhibited longer path lengths, whereas lateral trajectory deviations depended on the availability of visual feedback. Grasp success was higher for the visible hand during single-hand occlusion.

**Conclusion:**

Selective visual occlusion primarily influenced the later stages of bimanual reaching and grasping, while early movement planning remained comparatively robust. These findings demonstrate that limb-specific visual feedback contributes to coordinated bimanual behavior in a movement-phase-dependent manner and highlight the utility of immersive virtual reality for investigating the role of visual information during upper-limb coordination.

## 1 Introduction

Many activities of daily living require precise spatial and temporal coordination between the two hands to successfully reach, grasp, and manipulate objects. These coordinated actions rely on the integration of visual and proprioceptive information. Vision provides information about object location, size, orientation, and distance, while proprioception provides information about limb position and movement, allowing the nervous system to plan and control actions effectively (Jeannerod, 1984; Gentilucci et al., 1994; Flanagan and Johansson, 2003; Camponogara and Volcic, 2021).

Information about hand position and object location is used to guide both the reaching and grasp components of the movement, influencing movement timing, trajectories, and accuracy (Bingham et al., 2007; Rand et al., 2007; Volcic and Domini, 2016). Previous research has shown that reduced visual information leads to increased movement variability and greater reliance on proprioceptive feedback, whereas visual feedback supports online corrections that improve movement accuracy (Jeannerod, 1984; Elliott et al., 2001; Heath, 2005; Tremblay et al., 2013). Although these corrective processes often improve performance, they may also increase movement duration, particularly during the deceleration phase of the movement (Churchill et al., 2000; Mason, 2007b). Furthermore, even partial reductions in visual information about the hand have been shown to alter movement trajectories and reduce movement spatial efficiency (Bozzacchi et al., 2018).

If unimanual reach-to-grasp movements are influenced by visual feedback, bimanual movements present an even greater challenge for the sensorimotor system because visual attention must be coordinated across two limbs and often two targets simultaneously (Shea et al., 2012). Previous research has demonstrated that bimanual reach-to-grasp movements are associated with longer movement durations and greater coordination demands than comparable unimanual actions (Jackson et al., 1999). In addition, studies manipulating gaze and visual fixation have shown that visual attention influences temporal coordination between the hands, suggesting an important role for vision in modulating interlimb synchrony (Bruyn and Mason, 2009; Bingham et al., 2008). However, these studies manipulated gaze or task demands rather than directly manipulating visual feedback from the limbs themselves. Consequently, it remains unclear how visual feedback from each hand specifically contributes to bimanual coordination.

A major challenge in addressing this question has been the difficulty of selectively manipulating visual feedback while maintaining a natural task environment. Previous approaches have relied on mirrors, dark-room paradigms, fixation manipulations, or physical occlusion devices (Jeannerod, 1984; Bruyn and Mason, 2009; Bozzacchi et al., 2018; Volcic and Domini, 2016). Although informative, these methods often make it difficult to isolate visual feedback from the limbs while preserving visual information about the surrounding environment. As a result, it is unclear whether observed effects arise from the loss of visual information about the hand, the environment, or both. This experimental control is difficult to achieve using conventional methodologies because occluding one limb often alters visual information available about the surrounding environment.

Recent advances in virtual reality (VR) provide new opportunities to overcome these limitations. VR allows visual information to be manipulated independently for each limb while maintaining a controlled and immersive environment (Mason, 2007a; Mason et al., 2013; Nataraj et al., 2022; Guo et al., 2024). Previous work has demonstrated that reach-to-grasp behavior performed in VR closely resembles performance in physical environments, supporting its use as a tool for studying sensorimotor control (Furmanek et al., 2019). More importantly, VR makes it possible to selectively remove visual feedback from one or both limbs while preserving visual information about the environment and task goals.

Despite the importance of visual feedback for movement control, relatively little is known about how the availability of visual information from each limb independently influences bimanual reach-to-grasp performance. Understanding how this information contributes to bimanual coordination is important for advancing theories of sensorimotor integration and may also inform the development of VR-based assessment, training, and rehabilitation approaches.

The purpose of the present study was to examine how selective occlusion of visual feedback from one or both hands affects symmetric bimanual reach-to-grasp movements performed in a virtual reality environment. Previous research has demonstrated systematic differences between the dominant and non-dominant limbs during goal-directed movement, with the dominant hand typically associated with more efficient predictive control of movement dynamics and the non-dominant hand relying more heavily on sensory feedback for accurate execution (Peters, 1981; Sainburg and Kalakanis, 2000; Bagesteiro and Sainburg, 2002). Although hand dominance was not manipulated experimentally, all participants were right-hand dominant, allowing any interlimb differences to be interpreted within the context of these established functional asymmetries. Participants completed bimanual reaches under four visual feedback conditions: both hands visible (BV), left hand absent (LA), right hand absent (RA), and both hands absent (BA). Building on previous research demonstrating the importance of vision for movement coordination (Jackson et al., 1999; Bingham et al., 2008; Bruyn and Mason, 2009; Rand et al., 2007), we sought to determine how visual feedback from individual limbs influences the execution and coordination of bimanual reach-to-grasp movements, with emphasis on the reaching component, including movement timing, velocity, and trajectory control, while also examining grasp success as an overall task outcome.

We hypothesized that temporal coupling between the hands would be strongest when visual feedback from both hands was available (BV) and that selective visual occlusion would increase movement asynchrony, particularly when visual feedback from the left (non-dominant) hand was removed. We further predicted that visual occlusion would increase movement time and trajectory deviations due to a greater reliance on proprioceptive feedback and reduced opportunities for online correction. Finally, we expected the effects of visual occlusion to be most evident during the later stages of movement, when visual information is typically used to support online adjustments and interlimb coordination.

## 2 Materials and methods

### 2.1 Participants

Twenty healthy right-handed young adults (12 female, M = 23.4 ± 3.6 years) participated in this study. Participants were right-hand dominant (M = 79.75 ± 15.3 score) as determined by the Edinburgh Handedness Inventory (Oldfield, 1971; Veale, 2014) and had self-reported normal or corrected to normal vision. Exclusion criteria were neurological conditions, upper-limb, head, or spinal injuries. The Social Sciences Institutional Review Board at the University of Wisconsin-Madison approved the protocol (2022–1,411) before recruitment began. Participation was voluntary and involved one experimental session that was approximately 1 h long. Participants were provided $10 US as compensation for their time. Data from one participant was corrupt and thus excluded from all subsequent analyses.

### 2.2 Task

The experimental task consisted of bimanual reach-to-grasp movements in virtual reality (VR). Participants wore a fully immersive HTC VivePro™ VR headset and performed bimanual reaching and grasping movements to same-sized small virtual cubes (3 cm) under four different visual feedback conditions: (A) Graphic feedback about both hands visible (BV); (B) Graphic feedback about the right hand absent (RA); (C) Graphic feedback about the left hand absent (LA) and (D) Graphic feedback about both hands absent (BA). For each condition, participants completed three practice trials followed by twenty experimental trials, resulting in a total of 12 practice trials and 80 experimental trials across the four visual feedback conditions (92 trials in total). Condition A (both hands visible; BV) was always completed first. Based on pilot testing, participants appeared to require initial familiarization with the virtual environment and reach-to-grasp task under full visual feedback to reliably grasp the virtual cubes. During pilot testing, participants who began with the both-hands-absent (BA) condition failed to complete a sufficient number of successful reaches during the initial trials to permit reliable kinematic analyses. Therefore, in our experiment the BV condition was presented first for all participants to facilitate task familiarization before exposure to the visual occlusion conditions. The order of the remaining conditions (RA, LA, and BA) was counterbalanced across participants. The VR environment was a replica of the laboratory space.

**Figure 1:**
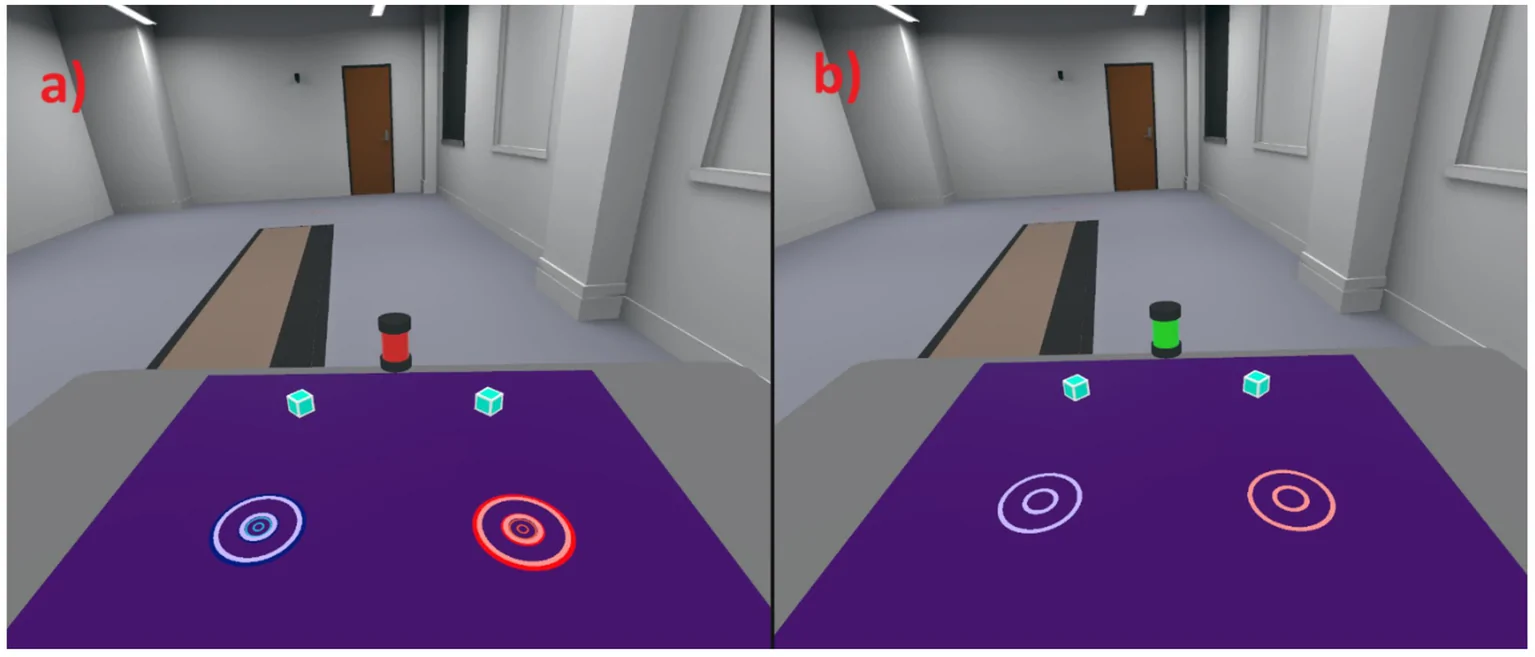
Participant view from VR headset during BA trial. **(a)** Left panel shows red light illuminated and concentric circles showing proper ready position. **(b)** Right panel shows green light on, and the participant has already started moving their hands towards the cubes (as evidenced by the concentric circles no longer being illuminated).

Participants sat, facing a table, on a height-adjustable, armless chair. The height of the chair was adjusted so that when participants rested their hands on the table surface, their elbows were flexed at 90 degrees. The chair was positioned at a distance from the table surface that allowed participants to comfortably reach the opposite edge of the table in the sagittal plane. Concentric circles in VR indicated the starting position of participants’ left (blue) and right (orange) index fingers. The starting positions were located 20.5 cm from the edge of the table. When participants correctly positioned their left and right index fingers over the designated start locations, both the inner and outer gaps between the concentric circles turned into a darker tone (see Figure 1a). If their fingers were misaligned, only the outer gap turned dark, indicating that a correction was needed before the next trial could begin. These visual cues enabled participants to accurately locate the starting positions with their index fingers, even in the absence of visual feedback of their hands.

A virtual stoplight was used as the “Go” signal. Participants were cued to begin reaching toward the target cube when the stoplight changed from red to green. The delay between the red and green lights was consistently 2,200 ms (see Figure 1b).

Participants were instructed to wait for the green “go” light and reach at a comfortable speed with both hands to grasp cubes with their index fingers and thumbs. Participants were also told to lift the cubes and bring them towards their chests after grasping them (to ensure a natural reaching and grasping behavior). The virtual cubes were placed 25 cm away from the starting position of both index fingers and rotated 45 degrees to facilitate a comfortable grasp posture (Mason et al., 2001). Participants were given 60 s breaks between conditions (every 23 trials) and were told they could request additional breaks if they felt disoriented, light-headed, or nauseated. No such instances occurred during testing and no participants requested additional breaks.

### 2.3 Apparatus

**Figure 2:**
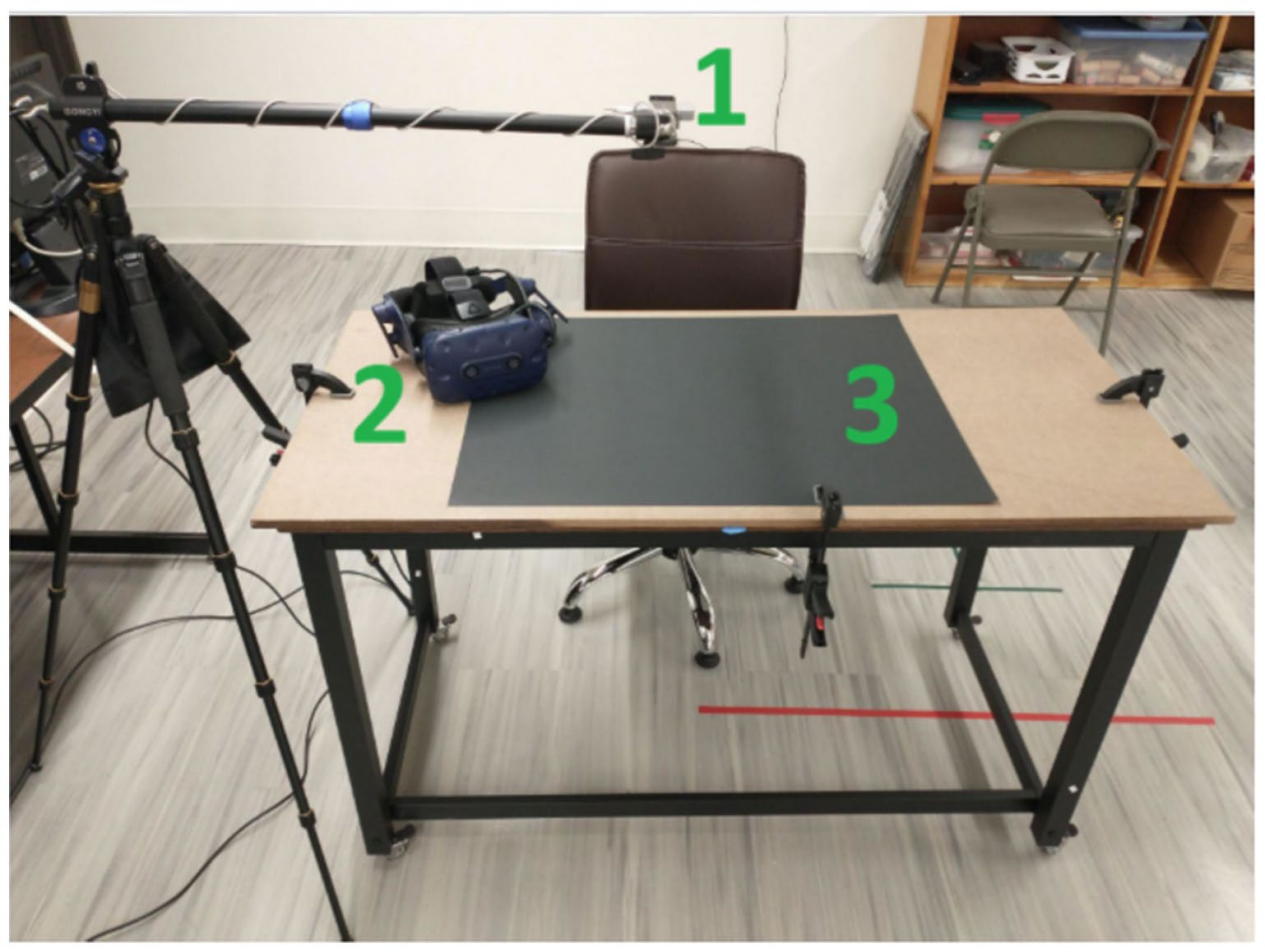
Experimental apparatus with numbered key components: (1) Mounted Leap Motion Hand Sensor; (2) HTC VivePro VR headset; (3) Black contrast surface.

**Figure 3:**
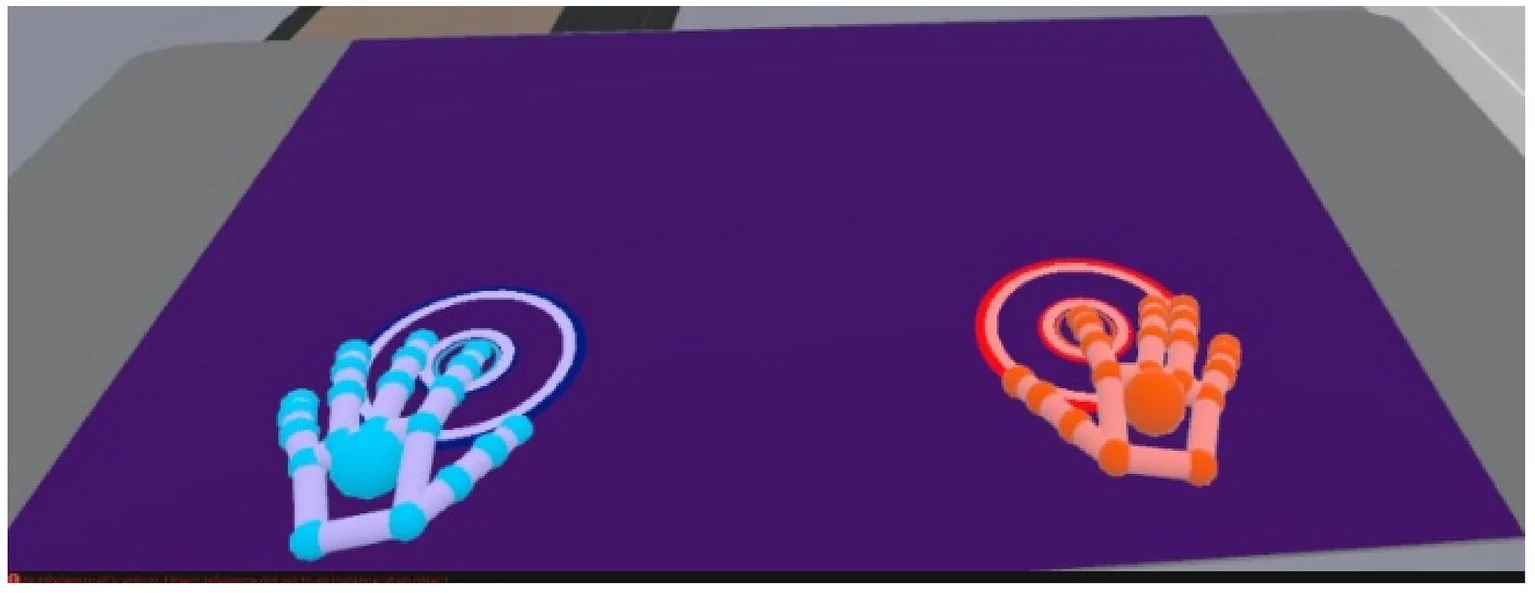
Graphic representation of hands inside VR environment in Unity 3D.

Figure 2 shows the experimental apparatus. A Leap Motion Hand Sensor device was mounted on a tripod and recorded hand movements at a frequency of 90 Hz. The table surface was covered with black paper to increase the contrast between participants’ limbs and the tabletop, allowing the Leap Motion camera to more accurately distinguish the hands from the background. To ensure spatial alignment across systems, an HTC Vive Tracker 3.0 was rigidly attached to the Leap Motion sensor using a custom-designed 3D-printed mount with precisely measured offsets. The tracker, Leap Motion sensor, and experimental table were all calibrated within a common coordinate system, ensuring that hand tracking data and the physical table were accurately represented within the same virtual space as the VR headset. Near real-time joint-segmented representations (see Figure 3) of the left and right hands were graphically displayed within the immersive virtual environment created in Unity 3D and presented via the HTC Vive Pro headset. The HTC Vive Pro has a refresh rate of 90 Hz, a 110° field of view (FOV), and a display resolution of 2,880 × 1,600 (1,440 × 1,600 per eye). The device was adjusted for each participant’s interpupillary distance (IPD) using measurements obtained from a Huanyu Digital Pupilometer (LY-9C).

### 2.4 Data analysis

We used customized software (KinSys, Eh? Soft) to analyze the three-dimensional position data recorded by the Leap Motion camera. All dependent measures were extracted via algorithms in KinSys and further verified visually to ensure accuracy. A 7 Hz low-pass, 2nd order Butterworth filter was used to smooth the data (Mason et al., 2013). Across the data from all 19 remaining participants and trials, a total of 6 out of 1748 trials were corrupt and had to be removed from further analysis. Because the primary objective of the present study was to examine how selective visual feedback influences the reaching component of bimanual reach-to-grasp movements, detailed kinematic analyses were restricted to wrist-based measures of reaching. Grasp performance was evaluated separately using task success rather than grasp kinematics.

Movement Initiation (MI) for each hand was defined as the point at which the resultant velocity of the wrist increased past a threshold of 5 mm/s (Mason and Bruyn, 2009). Movement End (ME) was defined as the local minimum (valley) of the resultant wrist velocity profile that fell below the velocity threshold (5 mm/s), corresponding to the time point at which forward (sagittal plane) velocity was closest to zero. All algorithmically identified MI and ME events were visually verified. In fewer than 1% of trials, the local minimum was manually selected when the predefined velocity threshold was not reached.

Movement times (MT) were subsequently calculated as the total time between MI and ME for each hand. Velocity data from all planes of motion and resultants were obtained by employing a 5-point differentiation estimate using the three-dimensional position data. Peak resultant velocity (PV) for the reach was determined for each wrist, as well as the time to reach peak velocity (TPV). Time from peak velocity (%TFPV) was calculated as a percent of MT occurring after peak velocity [(MT – TPV)/MT]*100. Path length (PL) was computed as the cumulative three-dimensional displacement of the wrist marker, obtained by summing the Euclidean distance between consecutive frames over the entire movement duration. Finally, peak lateral deviation (PLD) in cm was extracted for the wrist markers (maximum deviation of wrist from the straight line connecting the start position to the target object). Reaching kinematics were analyzed independently of grasp success. Thus, unsuccessful grasp attempts were retained for the kinematic analyses provided that they satisfied the predefined criteria for movement analysis. Because the reaching variables were defined prior to object contact, unsuccessful grasp attempts still represented valid reaching movements and were therefore considered appropriate for analysis of the reaching phase. Grasp success was analyzed separately as an overall behavioral outcome.

Grasping accuracy (success) was evaluated for each trial by determining whether participants successfully grasped and lifted the virtual object following the initial reach. Grasp success was recorded as a binary variable (successful/unsuccessful). A successful grasp occurred when index fingertips contacted the cubes within a predefined interaction region, causing the cubes to attach to the fingertips for the remainder of the movement according to the interaction rules implemented in Unity. This “sticky” interaction was intentionally implemented to provide a robust measure of successful object acquisition while minimizing unintended object drops resulting from limitations of the virtual physics simulation. Consequently, grasp success should be interpreted as successful completion of the grasping task for each hand separately rather than a measure of grasp quality or fine digit coordination.

Two Hand (left, right) * four Condition (BV, RA, LA, and BA) repeated-measures ANOVAs were employed to analyze MT, PV, TPV, %TFPV, PL, and PLD, with Condition as a within-subjects factor. Normality of the participant mean values was assessed using the Shapiro–Wilk test together with visual inspection of histograms and normal Q–Q plots. Although isolated departures from normality were identified for a small number of Hand * Condition combinations, visual inspection indicated only minor deviations attributable to single observations, and no extreme outliers requiring exclusion were identified. Given the robustness of repeated-measures ANOVA to minute departures from normality, parametric analyses were considered appropriate. Sphericity was assessed using Mauchly’s test. When the assumption of sphericity was violated, Greenhouse–Geisser corrections were applied. When significant main effects of Condition were observed, means were compared *post hoc* using pairwise comparisons with Bonferroni adjustment for multiple comparisons. When significant Hand * Condition interactions were found, these were further explored using simple main effects with Hand as the factor. Effect sizes are reported as partial eta squared (ηp^2^) and were interpreted according to values of 0.01, 0.06, and 0.14 representing small, medium, and large effects, respectively. Grasp success was analyzed using a two Hand * four Condition repeated-measures ANOVA with the same procedures applied. One-way repeated-measures ANOVAs with Condition as the factor were employed to analyze the timing differences between the hands for MI and ME. A post hoc sensitivity power analysis was conducted using G*Power 3.1 for the primary 2 (Hand) * 4 (Condition) repeated-measures ANOVAs. Assuming *α* = 0.05, power (1 − β) = 0.80, the final sample size (*N* = 19) provided sufficient power to detect effects of approximately *f* = 0.25, corresponding to a medium within-subject effect according to Cohen’s conventions. Thus, the study was adequately powered to detect medium-sized or larger within-subject effects. Statistical analyses were conducted using IBM SPSS Statistics 30.0.

## 3 Results

### 3.1 Movement timing

**Figure 4:**
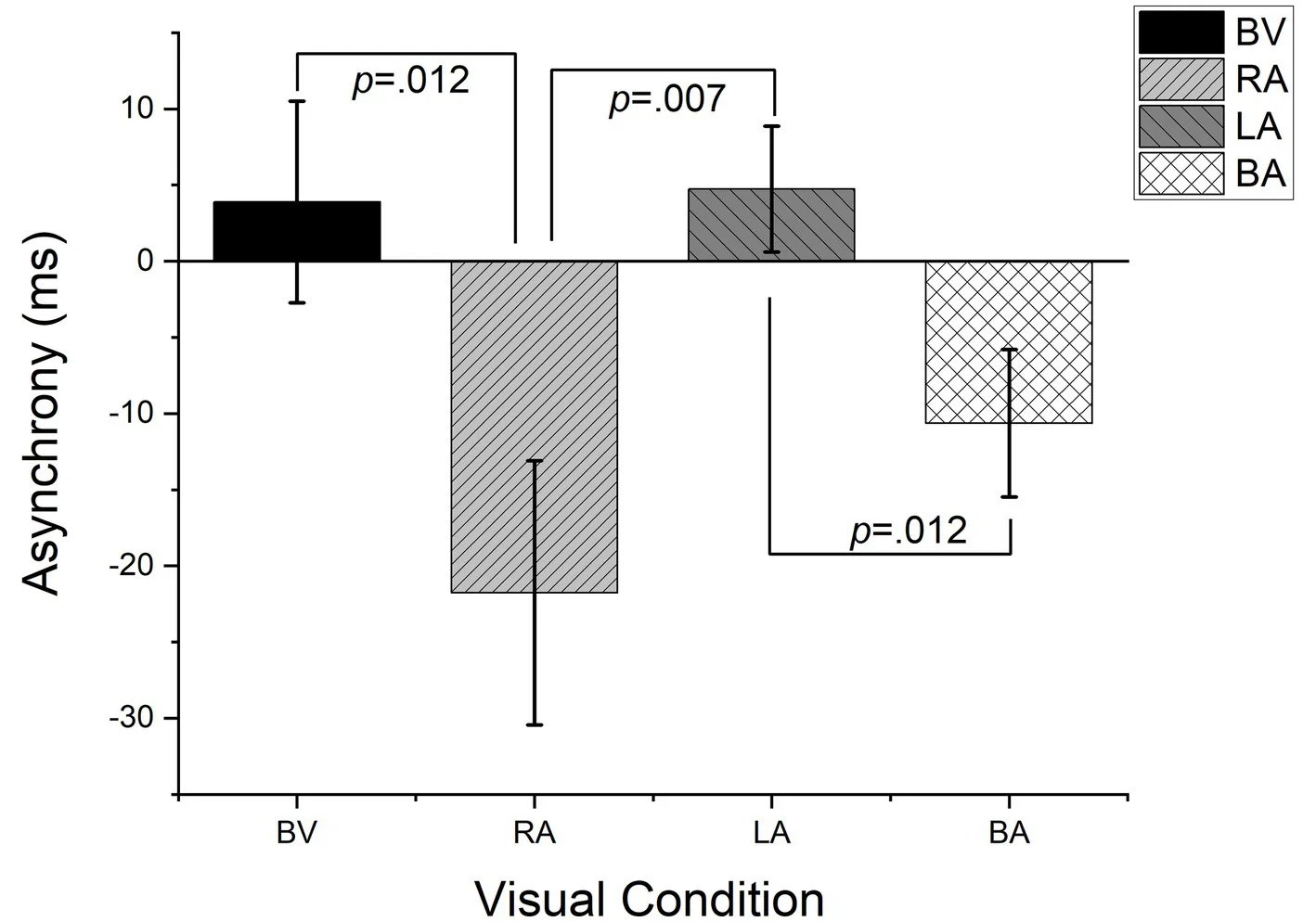
Mean movement end (ME) asynchrony (left − right) across visual feedback conditions. Positive values indicate that the left hand terminated later than the right hand, whereas negative values indicate that the right hand terminated later than the left. Error bars represent ± 1 SEM. Brackets indicate significant pairwise comparisons.

There were no effects of visual feedback condition on MI asynchrony (*p* = 0.129, ηp^2^ = 0.09). Across conditions, movement initiation remained highly synchronized between the hands, with the left hand beginning an average of only 6.6 ± 3 ms before the right. Conversely, there was a significant effect of visual feedback condition on ME asynchrony (*F*_(2.2, 35.3)_ = 4.815, *p* = 0.015, ηp^2^ = 0.211). In the BV and LA conditions, the left hand finished the movement after the right hand. In contrast, during the RA and BA conditions the right hand finished the movement after the left hand (see Figure 4).

**Figure 5:**
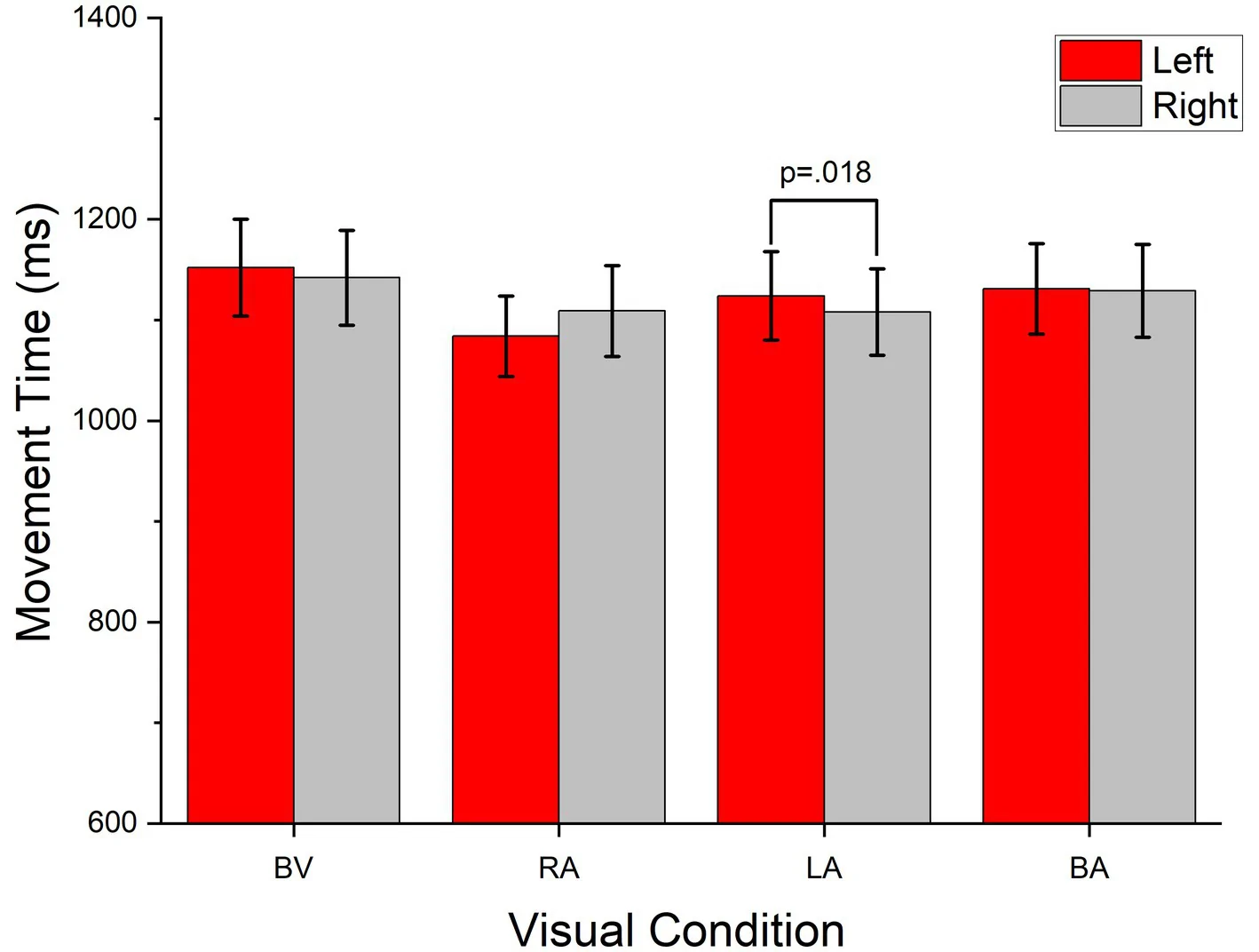
Mean movement time (MT) across visual feedback conditions for both hands. Error bars represent ± 1 SEM. Brackets indicate significant pairwise comparisons.

Although neither hand nor condition independently affected MT, a significant Hand * Condition interaction indicated that the effect of visual feedback differed between the hands (*F*_(2.2, 40.8)_ = 3.9, *p* = 0.025, ηp^2^ = 0.176). Specifically, participants had significantly longer movement times with their left hand compared to their right hand in the LA condition (*p* = 0.018) (Figure 5).

### 3.2 Movement velocity

**Figure 6:**
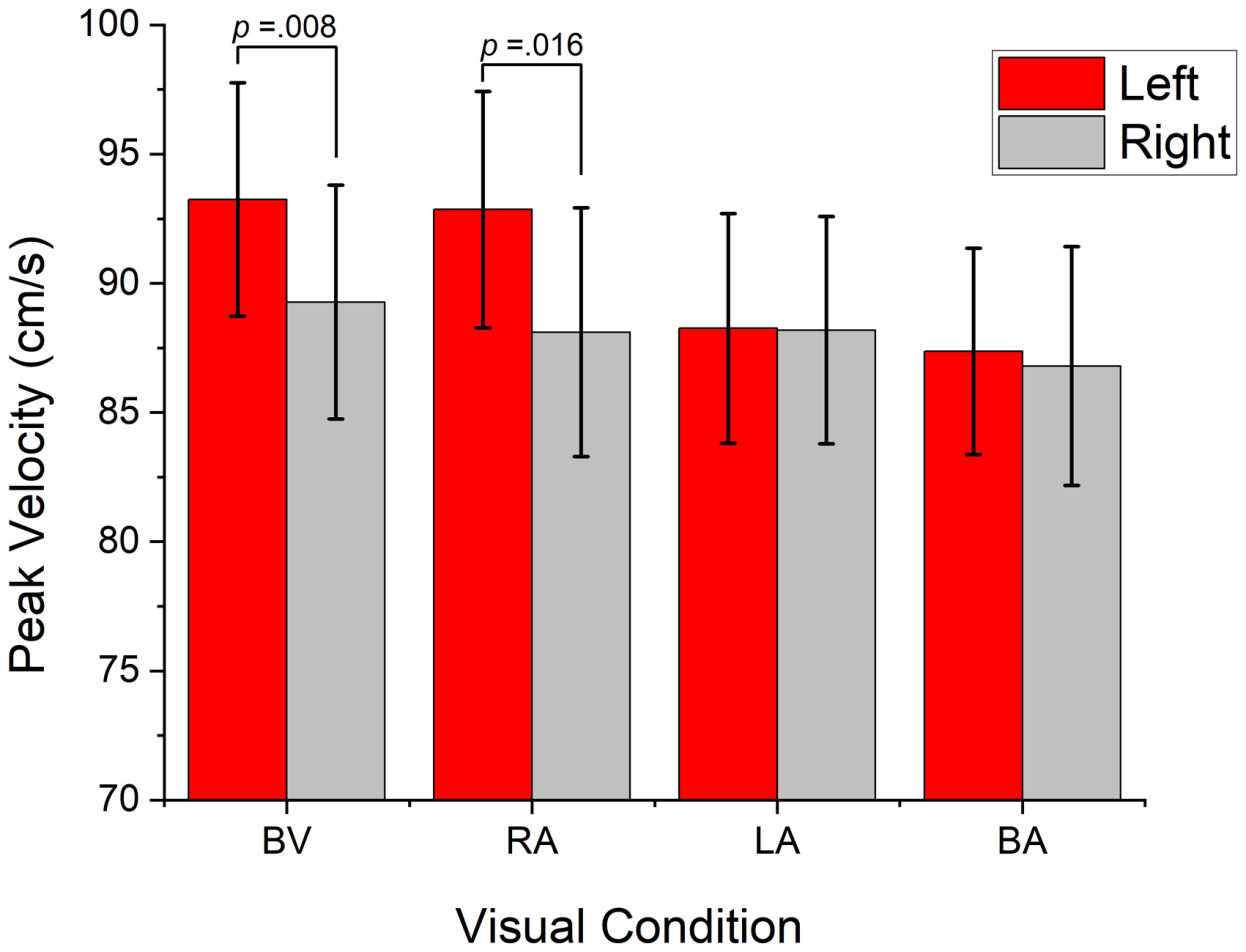
Mean peak velocity (PV) across visual feedback conditions for both hands. Error bars represent ± 1 SEM. Brackets indicate significant pairwise comparisons.

**Figure 7:**
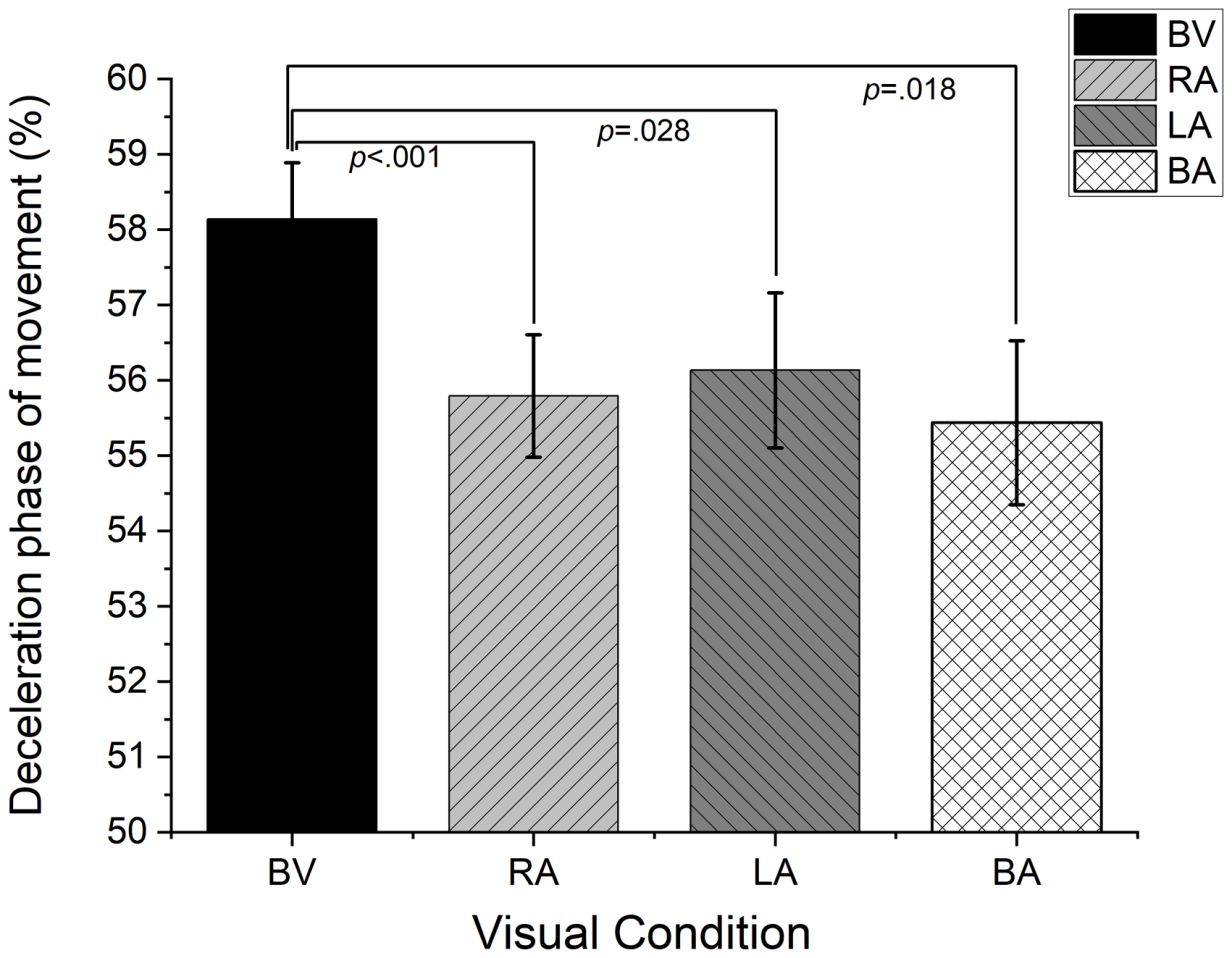
Mean percent time from peak velocity (%TFPV) across visual feedback conditions. %TFPV reflects the proportion of movement time spent in the deceleration phase following peak velocity. Error bars represent ± 1 SEM. Brackets indicate significant pairwise comparisons.

**Figure 8:**
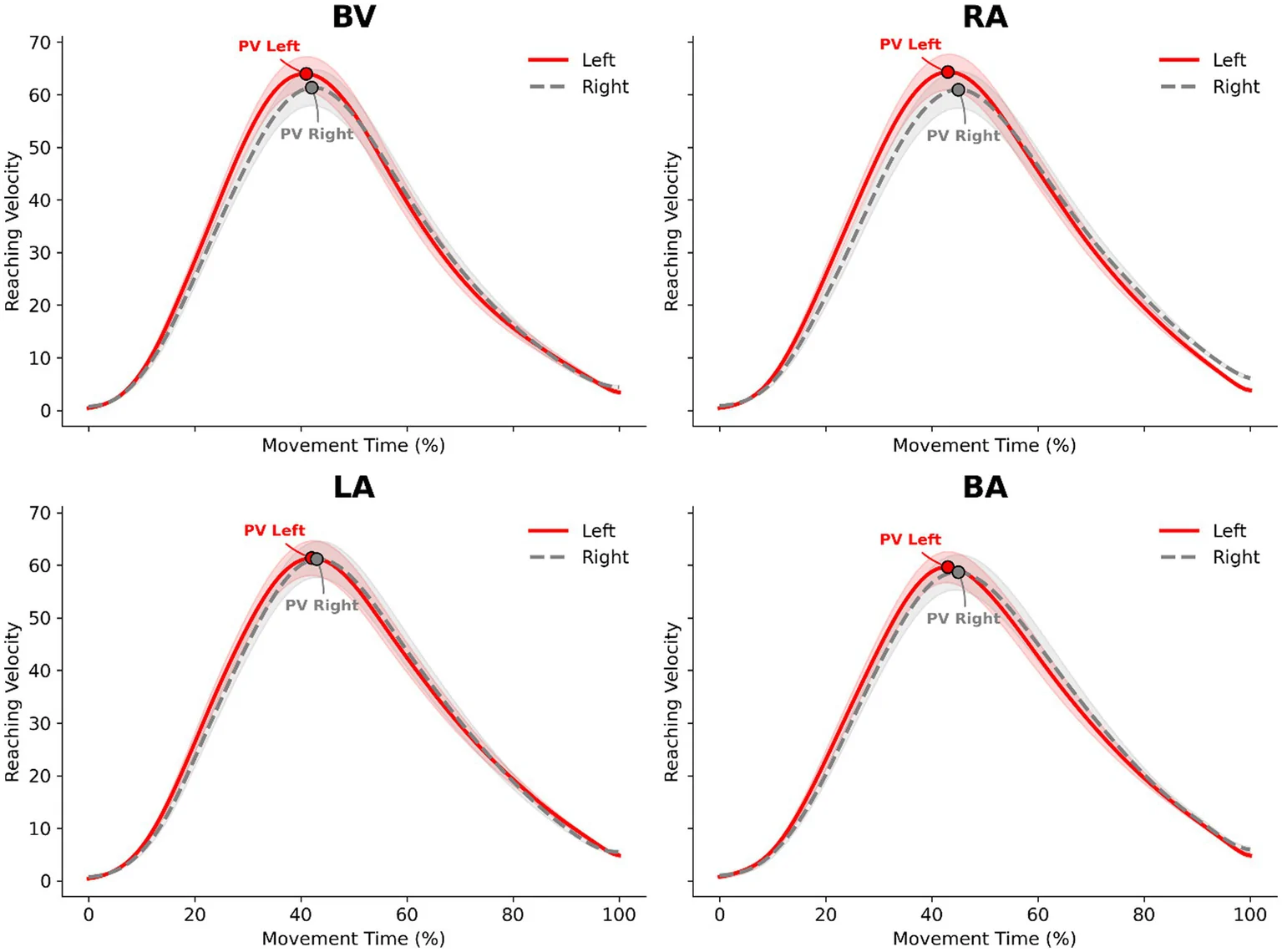
Mean resultant velocity profile (cm/s). Reaching velocity profiles were time-normalized to 100% movement duration and averaged within participants before computing the grand mean. Each panel represents a visual feedback condition, with left and right hand trajectories overlaid. Peak-velocity markers indicate the transition from the acceleration phase to the deceleration phase. Peak velocity was slightly higher for the left hand in the BV and RA conditions, whereas profiles were more similar between hands in the LA and BA conditions. The BV condition also showed a relatively prolonged deceleration phase. Shaded regions represent ±1 SEM across participants.

There were no main effects of visual feedback condition or hand for PV, but there was a significant Condition * Hand interaction (*F* (_3, 54_) = 6.11, *p* = 0.001, ηp^2^ = 0.252). Post-hoc analysis revealed that PV was significantly higher for the left hand compared to the right in two conditions (BV and RA) but similar in the other two conditions (LA and BA), (see Figure 6). There were no significant effects for TPV (*F*_(3, 54)_ = 1.09, *p* = 0.360, ηp^2^ = 0.057, grand mean = 364 ± 12 ms). However, there was a significant Condition main effect for %TFPV (*F*_(3, 54)_ = 4.82, *p* = 0.005, ηp^2^ = 0.211). Participants spent a greater proportion of movement time in the deceleration phase in the BV condition when compared to all other conditions (see Figure 7). Figure 8 illustrates the time-normalized reaching velocity profiles for both hands within each visual feedback condition. All conditions exhibited the typical bell-shaped velocity profile, with peak velocity occurring around mid-movement. Consistent with the statistical results, peak velocity was slightly higher for the left hand compared to the right in the BV and RA conditions, whereas profiles were more similar between hands in the LA and BA conditions. Additionally, the BV condition showed a more prolonged deceleration phase relative to the other conditions, aligning with the greater proportion of time spent after peak velocity.

### 3.3 Path length and lateral deviation

**Figure 9:**
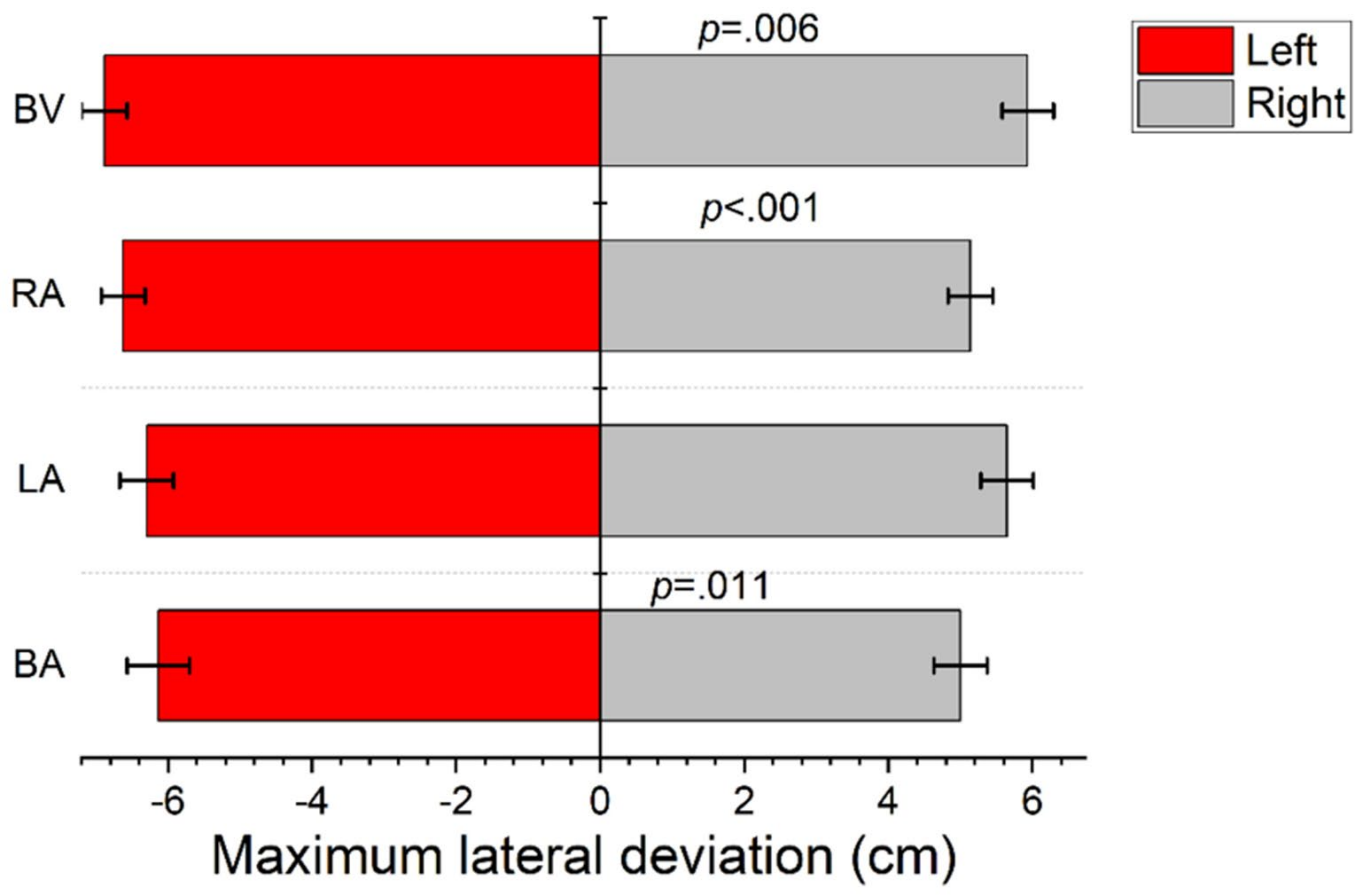
Mean peak lateral deviation (PLD) across visual feedback conditions. Positive values indicate deviation to the right from the sagittal plane, whereas negative values indicate deviations to the left. Error bars represent ± 1 SEM. *p*-values indicate significant between hand differences.

There was a significant main effect of hand for path length (PL) (*F* (_1, 18_) = 5.214, *p* = 0.035, ηp^2^ = 0.225). Post-hoc analysis showed that the left hand traveled significantly further (32.7 ± 0.5 cm) when compared to the right hand (31.9 ± 0.5 cm), regardless of visual condition. For wrist peak lateral deviation (PLD), there was a significant main effect of hand *(F*(_1, 18_) = 12.611, *p* = 0.002, ηp^2^ = 0.412), indicating that the left hand exhibited greater lateral deviations (6.4 ± 0.3 cm) than the right (5.4 ± 0.3 cm) across conditions. Follow-up analyses revealed significant differences between hands in the BV (*p* = 0.006), RA (*p* < 0.001), and BA (*p* = 0.011) conditions, with the left wrist exhibiting greater peak lateral deviation than the right. In contrast, the difference between hands was not significant in the LA condition, indicating that the typical asymmetry in lateral deviation was attenuated when visual feedback of the left hand was removed (Figure 9). PLD was modulated by the interaction between visual feedback condition and hand, with the left–right asymmetry being reduced when visual feedback of the left hand was selectively removed.

**Figure 10:**
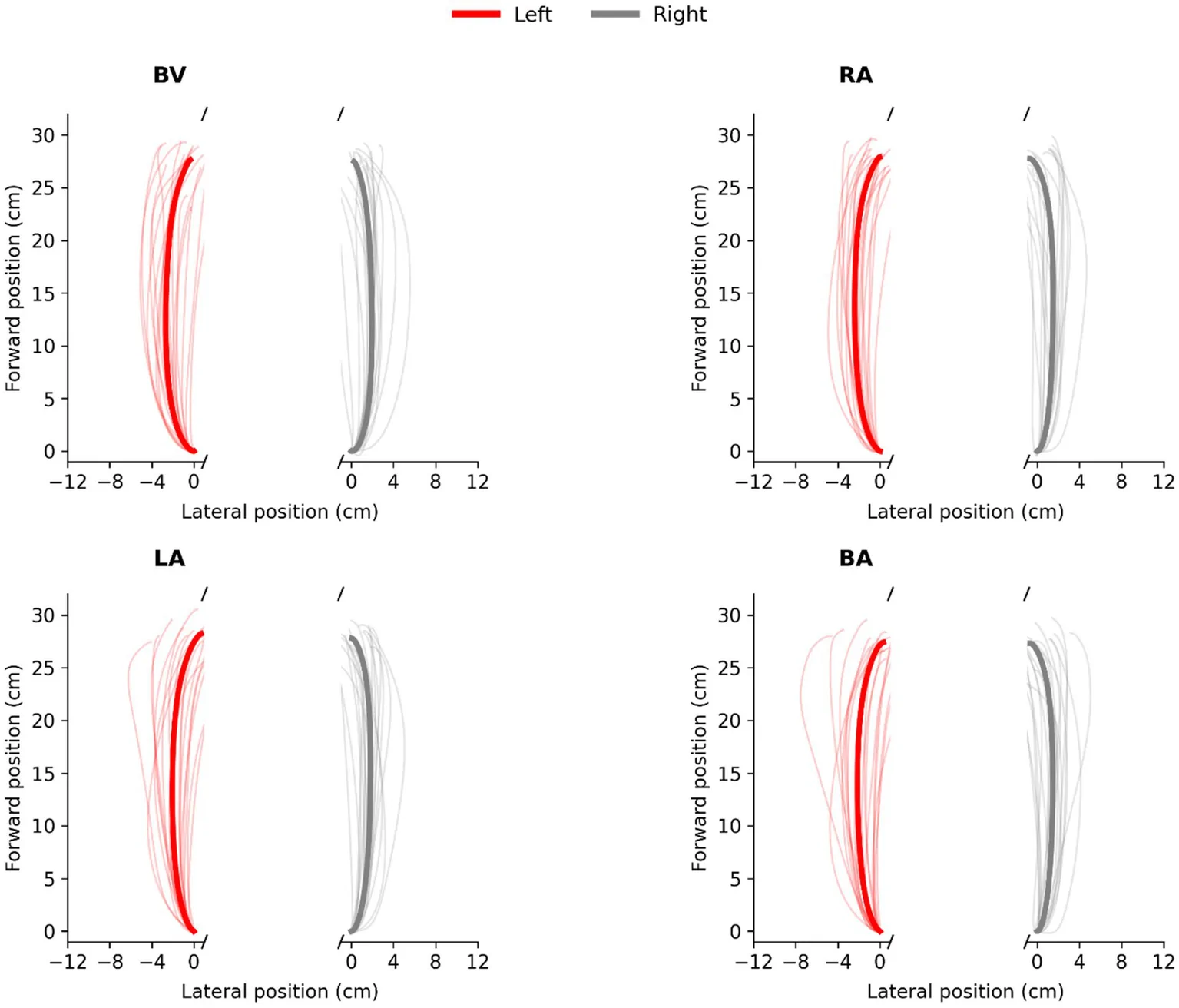
Mean normalized two-dimensional reaching trajectories. Transport trajectories were translated to a common starting position, time-normalized to 100% movement duration, and averaged within participants before computing the grand mean. Each panel represents one visual feedback condition (BV, RA, LA, and BA). Thin lines represent participant means, and thick lines represent the grand mean across participants. Left and right hand trajectories are displayed separately using a broken x-axis.

To facilitate visualization of these spatial differences, Figure 10 presents the mean normalized two-dimensional reaching trajectories for each visual feedback condition. Consistent with the PLD findings, the left hand generally exhibited greater lateral deviations from a straight reaching path than the right hand across conditions. The trajectories also illustrate that this left–right asymmetry was visually reduced in the LA condition, where reaching paths of the two hands appeared more similar. Although Figure 10 is descriptive, the grand mean trajectories represent well the PLD results by illustrating consistently greater lateral curvature of the left hand in BV, RA, and BA, with this asymmetry visibly reduced in LA.

### 3.4 Grasping accuracy

**Figure 11:**
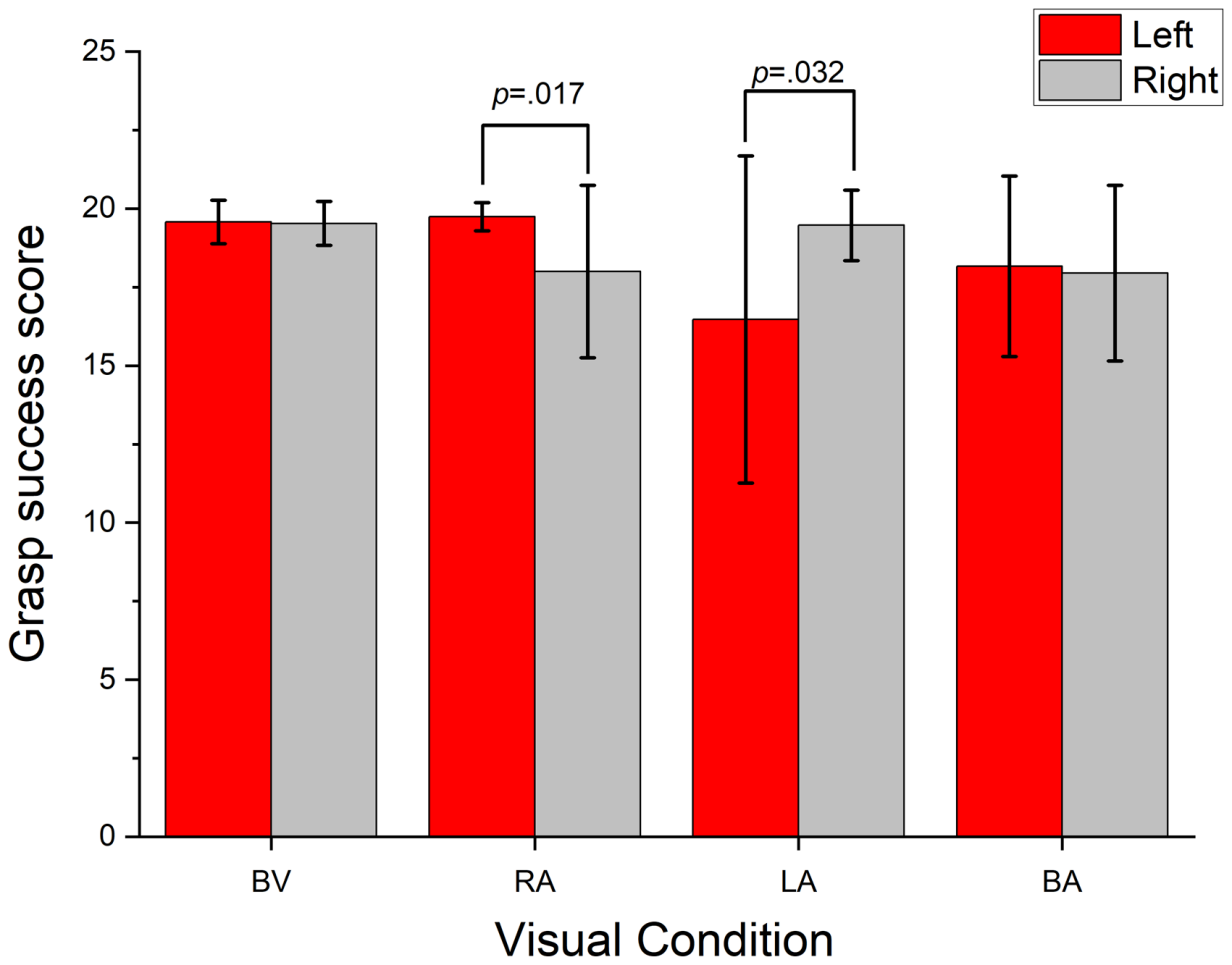
Grasping accuracy (success score) across visual feedback conditions. Maximum score possible was 20, minimum was 0. Grasp success reflects the number of times participants successfully grasped the cubes at the end of the reaching movement with their hands. Error bars represent ± 1 SEM.

Grasp success varied as a function of visual feedback condition, as reflected by a significant main effect of Condition (*F* (_2.1, 39.1_) = 4.56, *p* = 0.014, ηp^2^ = 0.202), but a conservative Bonferroni correction in the post-hoc analysis did not yield any significant differences between conditions. Nevertheless, there was also a significant interaction between visual feedback condition and hand (*F* (_1.3, 23.9_) = 5.59, *p* = 0.019, ηp^2^ = 0.237). Post-hoc analysis showed significant hand differences for the two single occlusion conditions. In both RA and LA conditions, the hands that were visible were more successful in grasping the objects than their not visible counterparts (see Figure 11).

## 4 Discussion

The aim of this study was to investigate how selective visual occlusion of one or both hands affects the coordination of symmetric bimanual reach-to-grasp movements in a VR environment. By precisely manipulating visual feedback using immersive VR and near real-time hand representations, we were able to examine how vision contributes to reaching kinematics and temporal coordination during bimanual reach-to-grasp movements while separately evaluating grasp success as an overall task outcome. Accordingly, the discussion is organized around the reaching kinematics, followed by consideration of grasp success as a behavioral outcome rather than a grasp-specific kinematic measure. Overall, the findings indicate that visual feedback primarily influenced the later stages of movement execution, while early feedforward components remained comparatively stable. Visual occlusion also altered spatial trajectory control and reduced grasp success for the occluded hand, highlighting the importance of limb-specific visual information during coordinated bimanual actions.

### 4.1 Temporal coordination and movement synchrony

Contrary to our hypothesis that the BV condition would present an advantage in temporal coupling at the onset of movement due to the availability of visual information, movement initiation was not significantly influenced by visual feedback. Across all conditions, participants initiated movements with both hands almost simultaneously, even when visual cues were absent. This pattern aligns with previous findings suggesting that movement onset in bimanual tasks relies more on internal timing mechanisms and proprioceptive calibration than on visual information (Srinivasan and Martin, 2010), and supports the idea that initiation is governed by centrally coordinated motor commands driving both limbs in parallel (Bingham et al., 2008).

In contrast, movement end (ME) asynchrony varied systematically across visual feedback conditions and was influenced by hand (right versus left), indicating that visual information becomes increasingly relevant in a limb-specific fashion as the movement unfolds. When visual feedback from the right (dominant) hand was unavailable (RA, BA), the right hand terminated the movement later. Conversely, when the right hand was visible (BV, LA), the left (non-dominant) hand lagged behind. This pattern suggests that visual feedback interacts with hand dominance during the later stages of movement execution. One possible explanation is that visual information may be preferentially used to support the temporal regulation of one limb over the other during movement termination. Previous work has shown that the direction of visual attention influences bimanual coordination (Bruyn and Mason, 2009), and therefore differences in attention allocation to one hand versus the other may have contributed to the observed movement-end asynchronies. However, because gaze behavior and visual attention were not directly measured in the present study, this interpretation remains speculative and should be considered a hypothesis for future investigation.

Importantly, the presence of ME asynchrony even in the full-vision (BV) condition extends prior work showing that visual information alone does not guarantee tightly synchronized bimanual movements. For example, Bruyn and Mason (2009) demonstrated that temporal coordination depends on how visual attention is allocated across the two targets, with fixation location influencing interlimb synchrony rather than eliminating asynchrony completely. Consistent with this view, our findings align with research showing that coordination becomes more variable with spatial separation and that vision does not fully eliminate interlimb timing differences (Bingham et al., 2008). Moreover, uneven visual allocation between limbs has been observed even under full-vision conditions (Fisk and Goodale, 1985), suggesting that perfect temporal coupling is generally not achieved.

The present findings are also consistent with lateralized control frameworks (Peters, 1981; Sainburg, 2002), which propose that the dominant limb is specialized for predictive control whereas the non-dominant limb relies more heavily on feedback-based processes. Within this framework, the ME asymmetries observed across visual feedback conditions may reflect an interaction between limb specialization and the allocation of visual information during the later stages of movement One possible explanation is that participants preferentially allocated visual attention to the visible hand when only one hand remained visible. When the dominant hand remained visible (BV and LA), attentional resources may have been preferentially directed toward that limb, allowing it to complete the movement slightly earlier while the non-dominant hand lagged behind. Conversely, when the dominant hand was occluded (RA) or when both hands were occluded (BA), this attentional advantage was absent, and movement termination was delayed for the dominant hand. However, because gaze behavior and visual attention were not measured directly, this interpretation is speculative. Future studies incorporating eye tracking will be important for determining whether differences in visual attention contribute to the temporal asymmetries observed during movement completion.

### 4.2 Movement timing and velocity

Movement time (MT), peak velocity (PV), and the percentage of time spent after peak velocity (%TFPV) provide complementary information about how visual feedback influences movement execution. Whereas PV reflected condition-dependent differences in early movement execution, MT and %TFPV demonstrated that visual feedback primarily influenced the later stages of movement, with MT increasing when visual feedback from a single hand was removed and %TFPV being longest in the fully visible condition (BV). When the left (non-dominant) hand was occluded (LA), MT increased despite similar PV compared with the visible right hand, suggesting that participants relied more heavily on online, feedback-based adjustments during movement execution rather than altering the initial motor plan. This interpretation is consistent with Rand et al. (2007), who showed that visual information supports movement path efficiency through online correction mechanisms, particularly for the non-dominant limb. The present findings further suggest that, when visual feedback from the non-dominant hand is unavailable, participants rely more heavily on proprioceptive information to maintain coordinated bimanual reaching, consistent with previous work demonstrating the complementary roles of vision and proprioception in online movement control (Bagesteiro et al., 2006; Sarlegna and Sainburg, 2009; Kasuga et al., 2022).

In conditions where the left hand was visible (RA and BV), the left hand exhibited higher peak velocities than the right hand. Because PV is generally considered to reflect feedforward aspects of movement planning, it is unlikely that these differences resulted from online use of visual feedback during the ballistic phase itself. Instead, the availability of visual feedback may have increased confidence in the planned movement or reduced uncertainty regarding task execution, resulting in more vigorous initial motor commands. Although visual feedback influenced early movement vigor in a hand-specific manner, its effects on movement timing became more evident during the later stages of movement, when online corrections and interlimb coordination demands were greatest.

The increase in MT observed with visual occlusion contrasts with previous unimanual studies reporting shorter movement times when visual feedback was removed (Jeannerod, 1984; Elliott et al., 2001). In those studies, the absence of vision was proposed to reduce online corrections, resulting in faster but less accurate movements. One possible explanation for the contrasting findings observed here is the additional coordination demands imposed by bimanual reaching. Because bimanual actions require the temporal coordination of both limbs (Swinnen, 2002; Bingham et al., 2008), participants may prolong the deceleration phase or delay movement termination under conditions of visual uncertainty to preserve coordinated performance, resulting in longer movement times despite similar peak velocities. Thus, the influence of visual feedback on movement time appears to depend not only on the availability of sensory information but also on the coordination demands of the task.

This interpretation is further supported by the %TFPV results, which showed the longest deceleration phase in the fully visible condition (BV), consistent with evidence that visual feedback selectively prolongs late adjustments during movement termination (Jeannerod, 1984; Mason, 2007a). When considered alongside the movement initiation and movement end findings, the present results suggest that the contribution of visual feedback differs across movement phases. Whereas early feedforward control remained comparatively robust, later stages of movement execution were more strongly influenced by limb-specific visual information. Furthermore, the non-dominant left hand exhibited greater sensitivity to visual feedback, showing higher peak velocities when visible and longer movement times when occluded, consistent with previous evidence that the non-dominant limb relies more heavily on sensory feedback during goal-directed actions (Bingham et al., 2008; Srinivasan and Martin, 2010; Sartori et al., 2011; Bozzacchi et al., 2018).

### 4.3 Trajectory deviations and movement efficiency

Trajectory deviations (PLD) revealed a significant interaction between visual feedback availability and hand, demonstrating that the influence of visual feedback on reaching trajectories differed between the two hands during bimanual reaching. Across most visual feedback conditions, the left (non-dominant) hand exhibited greater lateral deviations than the right, consistent with previous findings that the non-dominant limb produces less spatially efficient trajectories and relies more heavily on feedback-based control (Sainburg and Kalakanis, 2000; Bagesteiro and Sainburg, 2002; Tomlinson and Sainburg, 2012). Importantly, this asymmetry was attenuated when visual feedback from the left hand was selectively removed (LA), whereas significant interlimb differences remained in the other visual feedback conditions. The normalized grand mean trajectories shown in Figure 10 provide a qualitative illustration of this pattern, highlighting the greater lateral curvature of the left hand across most conditions and the reduction of this asymmetry during LA.

One possible explanation is that selective visual feedback altered how participants regulated movement trajectories during execution. The greater lateral deviations observed for the non-dominant hand are consistent with previous work demonstrating asymmetries in predictive control of limb dynamics (Sainburg and Kalakanis, 2000; Bagesteiro and Sainburg, 2002; Tomlinson and Sainburg, 2012). According to the Dynamic Dominance Hypothesis, the non-dominant limb is less effective at predicting and compensating for intersegmental dynamics, resulting in less spatially efficient trajectories. The present findings support this interpretation: the non-dominant hand consistently traveled longer paths across all visual conditions and exhibited greater lateral deviations in most conditions. Visual feedback therefore appears to have influenced how these asymmetries were expressed rather than altering the underlying limb-specific control strategies. Specifically, visual occlusion reduced the lateral deviation asymmetry without eliminating the underlying difference in movement efficiency between the hands. Together, the PL and PLD findings suggest that visual information interacts with pre-existing limb-specific control strategies to determine reaching trajectories during bimanual movements. However, because visual attention and gaze allocation were not measured directly, this interpretation remains speculative and should be confirmed in future studies.

These findings also highlight an important distinction between unimanual and bimanual control. In unimanual reaching, visual feedback primarily serves to reduce spatial error. In contrast, during bimanual reaching visual information must also be integrated with the requirement to coordinate the two limbs (Swinnen, 2002; Bingham et al., 2008). As a result, visual information cannot be used solely to optimize the trajectory of an individual limb but must also support coordination between the two limbs. Consequently, adjustments that improve the trajectory of one hand may be constrained by the need to preserve coordinated bimanual performance. This may explain why selective visual occlusion modulated trajectory deviations without eliminating the underlying asymmetry between the right and left limbs.

Although the present study did not quantify grasp kinematics, the grasp success results provide complementary information regarding overall task performance. The interaction between visual feedback condition and hand revealed a clear limb-specific pattern: in the single-occlusion conditions, the visible hand demonstrated greater grasp success than the occluded hand. This finding suggests that visual feedback facilitates successful task completion during grasping. However, because grasp-specific kinematic measures such as grip aperture, digit placement, and grasp formation were not collected, the mechanisms underlying this advantage cannot be determined from the present data. Although participants began each trial from standardized index-finger positions, the initial aperture between index fingers and thumbs was neither constrained nor quantified. Consequently, we cannot determine whether subtle differences in initial digit posture influenced the subsequent reaching kinematics or the coordination between the reaching and grasp components. Therefore, interpretations regarding grasp precision or contact accuracy should be made cautiously and warrant further investigation using more detailed grasp-related measures.

### 4.4 VR-specific considerations

The use of immersive VR allowed us to manipulate visual feedback with high precision. However, it also introduced unique perceptual constraints. As Harris et al. (2019) noted, reduced fields of view (FOV) and underestimated depth perception in VR may influence how participants interpret spatial information and perform visuomotor tasks. In our design, we prioritized a high refresh rate (90 Hz) and adjusted the immersive VR headset according to individual pupillary distance to minimize any possible effects of technological constraints. Furthermore, while our graphical hand representations supported real-time feedback, the absence of haptic feedback from the objects being grasped in our experimental setup is an important consideration. Studies by Camponogara and Volcic (2021) and Han et al. (2018) highlight the importance of tactile cues in refining movement execution and trajectory control. Future studies should incorporate passive or active haptics in order to clarify how visual and tactile systems support motor coordination in VR environments. However, despite these limitations, the present findings illustrate the experimental advantages of immersive VR by demonstrating that visual feedback from each limb can be manipulated independently while preserving visual information about the surrounding environment and task goals, an approach that is difficult to achieve using traditional laboratory paradigms.

### 4.5 Implications, limitations, and future directions

One important consideration when interpreting the present findings is that the both-hands-visible (BV) condition was always presented first to facilitate task familiarization. During pilot testing, participants who began with the both-hands-absent (BA) condition frequently failed to complete a sufficient number of successful reaches during the initial trials to permit reliable kinematic analyses, necessitating this design choice. Although participants completed practice trials before data collection, some degree of familiarization likely continued during the experimental session. Previous work has shown that performance in immersive virtual reality environments can continue to change with habituation, even after an initial familiarization period (Padilla et al., 2023). Motor learning theories similarly propose that individuals performing a novel motor task rely more heavily on visual and proprioceptive feedback during the early stages of skill acquisition (Magill and Anderson, 2021; Schmidt et al., 2019). To determine whether presenting the BV condition first introduced systematic familiarization effects or whether continued learning across the experimental session influenced the primary findings, we conducted supplementary repeated-measures analyses including trial block as a within-subject factor (see Supplementary material). These analyses indicated modest changes in performance across the experiment, together with a Condition × Block interaction that was primarily driven by the BV condition. Although movement time improved progressively across trial blocks in the BV condition, peak velocity remained stable throughout the experiment. Importantly, no Hand × Condition × Block interactions were observed for either variable, indicating that the principal effects of selective visual feedback remained stable despite continued task familiarization. Nevertheless, future studies should consider incorporating longer familiarization periods, objective performance stabilization criteria before data collection, and, where feasible, fully counterbalanced or randomized condition orders to further minimize potential learning effects and better isolate the contribution of visual feedback to bimanual coordination.

Moreover, trunk kinematics were not recorded, and participants were not externally restrained in order to preserve the natural coordination patterns that occur during unconstrained bimanual reaching, as external trunk support has been shown to alter the neuromuscular strategies used during reaching even when endpoint performance is preserved (Santamaria et al., 2018). Future studies incorporating whole-body motion analysis may help determine whether subtle trunk movements contribute to adaptations under selective visual occlusion. Finally, another possible limitation is that the sample was not balanced by sex, as participant recruitment reflected the available university population rather than stratified sampling. Although the present repeated-measures design minimizes the influence of between-subject variability and the study was not intended to examine sex-related differences, future studies with fully balanced samples may help determine whether the effects of selective visual feedback generalize similarly across sexes.

Overall, the present findings demonstrate that selective visual feedback influences coordinated bimanual reaching in a limb-specific manner, with its greatest effects occurring during the later phases of movement execution. Future studies should extend the present reaching-focused analyses by incorporating grasp-specific kinematic measures, including initial grip aperture, maximum grip aperture, grip aperture timing, and digit coordination, to determine whether the limb-specific effects observed during reaching also extend to the grasp component of the movement. Additional work combining immersive virtual reality with eye tracking, asymmetric task demands (e.g., different object sizes or reaching distances), and perturbation paradigms may further clarify how visual attention, sensory feedback, and limb dominance interact to support coordinated bimanual behavior. Such studies may also contribute to the development of virtual reality-based assessment and rehabilitation strategies that target coordinated upper-limb function.

## Data Availability

The raw data supporting the conclusions of this article will be made available by the authors, without undue reservation.
