# Supplementary material for: Effects of limb-specific visual occlusion on bimanual reach-to-grasp coordination in an immersive virtual reality environment
Source: Front Psychol. 2026 Aug 19;17:1884831. doi: 10.3389/fpsyg.2026.1884831 (PMC13533745; doi:10.3389/fpsyg.2026.1884831)
Supplement: Supplementary file 1 [file Supplementary_material_1.DOCX]

**Supplementary Analysis: Task Familiarization**

1. **Movement Time**

To evaluate whether task familiarization influenced performance across the experimental session, movement time (MT) was reanalyzed using a 2 (Hand) × 4 (Condition) × 4 (Block) repeated-measures ANOVA, in which the experimental trials within each condition were divided into four consecutive blocks of five trials. A significant main effect of Block was observed (*F* (_2.3, 41.6_) = 5.40, p = .006, ηp² = .231). Pairwise comparisons showed that MT was significantly shorter in Block 3 than in Block 1 (p = .011), whereas no other block comparisons reached significance. However, we should interpret this in the light of a significant Condition × Block interaction (*F* (_2.8, 50.7_) = 4.46, p = .008, ηp² = .199). Follow-up pairwise comparisons indicated that significant differences between blocks occurred only in the both-hands-visible (BV) condition (see Figure S1). Specifically, MT was shorter in Block 2 than Block 1 (p < .001), Block 3 than Block 1 (p = .007), and Block 4 than Block 1 (p = .014). No significant differences between trial blocks were observed in the remaining visual feedback conditions (all p > .05).

Importantly, neither the Hand × Block interaction (p = .251) nor the Hand × Condition × Block interaction (p = .462) was significant. Furthermore, the original Hand × Condition interaction remained significant after including trial block in the model (*F* (_2.2, 40.1_) = 3.79, p = .027, ηp² = .174), indicating that the principal effects of selective visual feedback were not explained by progressive task familiarization.

**Supplementary Figure S1 -**  *Mean movement time across four consecutive experimental blocks for each visual feedback condition.* Each block consisted of five trials. Error bars represent ±1 SEM. Although movement time decreased during the early stages of the experiment, this effect was primarily driven by the both-hands-visible (BV) condition, whereas performance remained relatively stable across the remaining visual feedback conditions.

2. **Peak Velocity**

Peak velocity (PV) was similarly reanalyzed using a 2 (Hand) × 4 (Condition) × 4 (Block) repeated-measures ANOVA. In contrast to MT, there was no significant main effect of Block (p= .124). A significant Condition × Block interaction was observed (*F* (_2.6, 46.8_) = 5.35, p = .003, ηp² = .229). Follow-up pairwise comparisons indicated that significant differences between trial blocks occurred only in BV condition. Specifically, PV was greater in Block 2 than Block 1 (p = .015), Block 3 than Block 1 (p < .001), and Block 4 than Block 1 (p < .001). No significant differences between trial blocks were observed in the remaining visual feedback conditions (all p > .05)(see Figure S2). Neither the Hand × Block interaction (p = .930) nor the Hand × Condition × Block interaction (p = .423) was significant. Furthermore, the original Hand × Condition interaction remained significant after including trial block in the model (*F* (_2.4, 43.2_) = 6.06, p = .003, ηp² = .252), indicating that the principal effects of selective visual feedback on peak velocity were not explained by progressive task familiarization.

**Supplementary Figure S2 -**  *Mean peak velocity across four consecutive experimental blocks for each visual feedback condition.* Each block consisted of five trials. Error bars represent ±1 SEM. Peak velocity remained relatively stable across the experimental session, with only modest condition-dependent changes over time.

3. **Conclusion**

Taken together, these supplementary analyses indicate modest task familiarization effects, primarily during the both-hands-visible (BV) condition. Importantly, the absence of significant Hand × Condition × Block interactions for both MT and PV, plus the persistence of the original Hand × Condition interactions, demonstrates that the principal effects of selective visual feedback remained stable throughout the experiment and were not attributable to progressive task familiarization.
